# A Case of Pleuro-Pneumonia, Associated with Intermittent Fever

**Published:** 1868

**Authors:** Wm. O’Daniel

**Affiliations:** Marion, Ga.


ATLANTA

### Medical and Surgical Journal.

NEW SERIES.

Vol. IXA. MAY and JUNE, 1868. No. 2.

### ORIGINAL COMMUNICATIONS.

### ARTICLE I.

A Case of Pleuro-pneumonia, Associated with Intermittent Fever. By Wm. O'Daniel, M. D., of Marion, Ga.

On the 20th of December last, at 8 o'clock, p. m., I was called to see Peter Y——, a colored boy about fourteen years old.

Upon inquiry, I found that he had had several attacks of Intermittent fever during the summer and fall months. I examined the patient carefully, and found the following symptoms existing:

Spleen and liver very much enlarged, crepitant rhoncus; bronchial respiration, though not very distinct; skin hot and dry; circulation 120 per minute, with considerable volume; bowels constipated, and very much swollen; tongue furred; very acute pain on either side near the nipples, and also under the right scapula; constant cough, attended with slight expectoration of a bloody character; respiration hurried; patient rests most comfortably on back.

Had a chill on the 15th of December at night, followed by high febrile excitement, though the fever abated by next evening. Had another on the 17th at night, followed as before by high fever, though it did not abate as it did before. It was then thought by the employer of the child's father, who is a very intelligent gentleman indeed, to be a case of simple Intermittent fever; consequently, he endeavored to meet the next paroxysm with quinine, which failed to have the desired effect, and I was, as I before said, called to see him on the 20th, and diagnosticated the case as above, and employed the following treatment, to be commenced at 9 o'clock, P. M.,

℞—Hyd. chlor. mit.
Pulv. rhei. *aa.* grs., x.

which evacuated the bowels freely by morning. I then employed the following:

℞—Potas. nit., grs. xxx.
Antim. et potas. tart., grs. j.

Mix et ft. chart. No. 5. One to be given every 3 hours.

Called to see patient on the evening of the 21st, and found the circulation 100 per minute; bronchial respiration; dullness on percussion; perspiring freely; pain in the breast more lancinating than on the day before.

I applied a large blister over the region of the lungs, and prescribed—

℞—Quinia, grs. xv.
Potas. nit., grs. xx.
Pulv. doveri, grs. xij.
Antim. et potas. tart., grs. ss.

Mix et ft. chart. No. 3. One every 3 hours, to be commenced at 12 o'clock at night.

Called at 9 o'clock on the morning of the 22d, and found circulation 90 per minute; expectoration rusty; sputa free; resting well.

Prescribed—

℞—Potas. nit., grs. xxx.
Antim. et potas. tart., grs. j.

Mix and divide into chart No. 5. One to be given every 3 hours.

℞—Quinia, grs. xv.
Pulv. doveri, grs. xij.

Mix et ft. chart. No. 3. One to be given every 3 hours, to be commenced at 12 o'clock at night.

Called again at 9 o'clock on the morning of the 23d, and found him expectorating freely. Circulation 90 per minute, and doing well.

Prescribed—

℞—Potas. nit., grs. xxx.
Antim. et potas. tart., grs. j.
Pulv. doveri, grs. xv.

Mix et ft. chart. No. 5. One to be given every 3 hours. Advised suitable nourishment, and brandy and water, pro re nata.

Called on the 24th. Found patient discharging sputa freely; able to sit up in bed a short while at a time; considered him much better, and continued treatment.

Called again on the 25th, and found him still improving; discharge from lungs considerably less; continued treatment.

Called on the 27th, and found him still improving; able to walk about the room. Continued treatment, and dismissed the case.

On the 2d of January I was called to see patient in great haste. Upon my arrival, I found him dead. I was reliably informed that he continued to improve, and got able to be up most of his time, and, unexpectedly to every one, was seized by a convulsion, and died in the 12th one, 3 hours after first attacked by them.

Autopsy 6 hours after death revealed the following: Traces of hepitization of base of both lungs; thorax and right lung adhered, which verifies the existence of pleuritis. Adhesion of pericardium, with considerable effusion.

